# Image segmentation using active contours with modified convolutional virtual electric field external force with an edge-stopping function

**DOI:** 10.1371/journal.pone.0230581

**Published:** 2020-03-26

**Authors:** Ke Cheng, Tianfeng Xiao, Qingfang Chen, Yuanquan Wang

**Affiliations:** 1 School of Computer, Jiangsu University of Science and Technology 1, Zhenjiang, China; 2 School of Electronics and Information, Jiangsu University of Science and Technology 2, Zhenjiang, China; Zhejiang University, CHINA

## Abstract

The gradient vector flow (GVF) is an effective external force to deform the active contours. However, it suffers from high computational cost. For efficiency, the virtual electric field (VEF) model has been proposed, which can be implemented in real time thanks to fast Fourier transform (FFT). The VEF model has large capture range and low computation cost, but it has the limitations of sensitivity to noise and leakage on weak edge. The recently proposed CONVEF (Convolutional Virtual Electric Field) model takes the VEF model as a convolutional operation and employed another convolution kernel to overcome the drawbacks of the VEF model. In this paper, we employ an edge stopping function similar to that in the anisotropic diffusion to further improve the CONVEF model, and the proposed model is referred to as MCONVEF (Modified CONVEF) model. In addition, a piecewise constant approximation algorithm is borrowed to accelerate the calculation of the MCONVEF model. The proposed MCONVEF model is compared with the GVF and VEF models, and the experimental results are presented to demonstrate its superiority in terms of noise robustness, weak edge preserving and deep concavity convergence.

## 1. Introduction

Image segmentation is a fundamental problem in computer vision [1,2] and the active contour, or snake model [3,4], dominates this community in the past thirty years. The active contour model transforms the image segmentation issue into an energy minimization problem by minimizing an integration of the internal energy of a curve and its external energy. The internal energy comes from the continuity and smoothness of a curve and the external energy is derived from the edge map of an image. However, due to the local nature of the gradient of the edge map, the traditional snake model suffers from initialization sensitivity and concavity handicap. To note, the deep learning based methods launch an upsurge of image segmentation at present, [5,6] present two great examples among others.

In order to overcome the drawbacks of the traditional snake model, Xu and Prince proposed the gradient vector flow (GVF) model that is one of the most successful external forces to drive the snake contour [7,8]. The GVF model diffuses the gradient vector from the object boundary to the rest of the image and enlarges the capture range while suppresses the influence of noise to some degree. Due to its effectiveness, the GVF model has been the focus of research for a long time and there are many variants aiming at improving the performance of the GVF further or extending its applications. For example, the harmonic gradient vector flow (HGVF) employs the curl and divergence of the gradient vector field as constraint [9], minimal surface [10] and harmonic surface [11] are also employed to reformulate the gradient vector flow. Other examples include the BVF [12], NGVF [13], NBGVF [14], MGVF [15], EPGVF [16], CN-GGVF [17], DDGVF [18], 4DGVF [19], guided filter based GVF [20] and GVFOM [21]. Very recently, there are several interesting works focusing on the initialization of the GVF snake model, which includes the phase portrait analysis [22], fusion of the conventional US image with elasticity and Doppler images [23], and walking particles [24,25].

Although the GVF model is effective, it has to solve two PDEs (partial differential equations) throughout the entire image and is short of efficiency. In order to overcome the inefficiency of the GVF model, several fast algorithms have been proposed, for example, the multigrid method based GVF [26], augmented Lagrangian method based GVF [27], and efficient numerical schemes for the GVF [28]. There are two impressive works approximating the GVF, which are based on convolution and FFT and can be implemented in real-time, i.e., the virtual electric field (VEF) [29] and the gradient vector convolution [30]. In the VEF model, each pixel in an image is considered as an electron, and all the pixels in the image will generate a virtual electric field. The VEF can be implemented in real time by using the fast Fourier transform (FFT) after some manipulations. The VEF model possesses some desirable properties of the GVF model such as large capture range and concavity convergence, and has low computation cost, however, there is room for improvement on noise robustness and weak edge preserving. The recently proposed CONVEF model extends the VEF model by using another convolution kernel and can preserve weak edges to some degree [31].

However, the convolution kernel in the CONVEF model is still linear, and the effectiveness can be improved further. In this paper, an edge stopping function similar to that in the anisotropic diffusion [32] is adopted to further improve the CONVEF model on preserving weak edges. The proposed model is referred to as MCONVEF (Modified CONVEF). A piecewise linear approximation method is borrowed to accelerate the calculation of the MCONVEF model [33], so that the MCONVEF model can also be implemented by using FFT(fast Fourier transform), and the computation cost of the MCONVEF is comparable to that of the CONVEF and VEF models. To note, the CONVEF [31] and the BVEF [1] models are related to the VEF model, both of the two models are different from the MCONVEF model. The CONVEF model employs a linear kernel similar to the VEF model, see Eq (9), whist the MCONVEF employs an edge stopping function so that the kernel is nonlinear, see Eq (10). When compared with the BVEF model [1], there are two different points, first, there is a scale factor in the MCONVEF model to resist noise, and then, an edge-stopping function in the anisotropic diffusion is borrowed to nonlinearize the kernel, which is different from the Gaussian function employed in the BVEF model. So, the MCONVEF snake possesses more properties than the BVEF snakes, such as noise robustness, and concavity convergence, as shown in the experiments. In order to simultaneously extract the cardiac geometry and kinematics from cine tomographic medical image sequence [34], Liu et al proposed a novel active region model, where each node spatiotemporally evolves under the influences of the node-dependent imaging data, temporal consistency models of the tissue geometry and kinematics, and statistical priors of the myocardium spatial distributions. The external force is different from the proposed MCONVEF, which comes from four parts: edginess, prior, shape and temporal constraint while the MCONVEF is derived from convolution.

The remainder of this paper is organized as follows: in the next section, the snake, GVF, VEF and CONVEF models are briefly reviewed. The proposed MCONVEF model is described in detail in Section 3. In Section 4, various experiments are presented to demonstrate the properties of the proposed MCONVEF model and conclusion is drawn in Section 5.

## 2. Backgrounds: Snakes, GVF, VEF and CONVEF

### 2.1. Snakes: Active contours [3]

A snake is an elastic curve *c*(*s*) = (*x*(*s*),*y*(*s*)),*s*∈[0,1], that moves through the spatial domain of an image to minimize the following energy functional,

$$E_{Snake}=\int_{0}^{1}\left[\frac{1}{2}(\alpha {\left|c\prime (s)\right|}^{2}+\beta {\left|c\prime \prime (s)\right|}^{2})+\nabla E_{ext}(c)\right]$$ (1)

Where *c*′(*s*) and *c*″(*s*) are first and second derivatives of *c*(*s*) with respect to *s*, *α* and *β* are weighting parameters. The external energy *E*_*ext*_ is derived from the image data and takes smallest values at boundaries. The typical external force for gray-value image is *E*_*ext*_ = −|∇*G*_*σ*_⊗**I**|^2^, where *G*_*σ*_ is the Gaussian kernel of standard deviation *σ*. A snake that minimizes *E*_*Snake*_ must satisfy the Euler equation,

$$\alpha c^{\prime \prime }(s)-\beta c^{\prime \prime \prime \prime }(s)-\nabla E_{ext}=0$$ (2)

This can be viewed as a force balance equation of internal and external forces,

$$F_{int}+F_{ext}=0$$ (3)

where *F*_*int*_ = *αc*″(*s*)−*βc*″″(*s*) and *F*_*ext*_ = −∇*E*_*ext*_. The internal force *F*_*int*_ makes the curve to be continuous and smooth while the external force *F*_*ext*_ attracts the curve to the desired features of the image.

### 2.2. GVF: Gradient Vector Flow [7,8]

In order to overcome the ‘myopia’ nature of the traditional external force based on the edge map of the image, Xu and Prince proposed the Gradient Vector Flow (GVF) external force [7]. The GVF is a vector field **v**(*x*,*y*) = [*u*(*x*,*y*), *v*(*x*,*y*)] obtained by minimizing the following energy functional,

$$E_{GVF}=\iint \mu (u_{x}^{2}+u_{y}^{2}+v_{x}^{2}+v_{y}^{2})+{\left|\nabla f\right|}^{2}{\left|v-\nabla f\right|}^{2}dxdy$$ (4)

where *f* is the edge map of an image, usually, *f* = |∇*G*_σ_*I|; *μ* is a regularization parameter governing the tradeoff between the smoothness constraint and the fidelity term in (4). Using the calculus of variations, to minimize the *E*_*GVF*_ is converted to solve the following Euler-Lagrange equations,

$$\left\{ \begin{array}{l} \partial u/\partial t=\mu \nabla^{2}u-{\left|\nabla f\right|}^{2}(u-f_{x})\\ \partial v/\partial t=\mu \nabla^{2}v-{\left|\nabla f\right|}^{2}(v-f_{y}) \end{array}\right.$$ (5)

where ∇^2^ is the Laplacian operator.

### 2.3. VEF: Virtual Electric Field [29]

Since the GVF model needs to solve the partial differential Eq in (5) in an iterative manner, it is very time-consuming and hinders the application of the GVF model in real-time scenarios. To address this issue, Park and Chung presented the virtual electric model (VEF) [29] model by taking each pixel in image as an electron with the charge being the magnitude of the edge of the image, and the virtual electric field at (*x*_0_,*y*_0_) is derived from the sum of all other electrons in region D around (*x*_0_,*y*_0_), which is expressed as,

$$E_{VEF}\left(x_{0},y_{0}\right)=\sum_{\left(x,y\right)\in D}\left(\left(x_{0}-x\right)/d^{3},\left(y_{0}-y\right)/d^{3}\right)\cdot f\left(x,y\right)$$ (6)

where $d=\sqrt{{\left(x_{0}-x\right)}^{2}+{\left(y_{0}-y\right)}^{2}}$ *D* = {(*x*,*y*)|−*t*≤*x*_0_−*x*≤*t*, −*t*≤*y*_0_−*y*≤*t*}, *f*, is the magnitude of the edge image of an image. In order to apply the fast Fourier transform (FFT) to the calculation, the VEF model in (6) is usually written as a form of convolution as follow,

$$E_{VEF}\left(x,y\right)=K\left(x,y\right)⊗f\left(x,y\right)$$ (7)

where ⊗ denotes the convolution operation and *K*(*x*,*y*) is the convolution kernel with the following form,

$$K\left(x,y\right)=\left(-x/d^{3},-y/d^{3}\right)$$ (8)

where $d=\sqrt{x^{2}+y^{2}}$ is the distance. Besides the efficiency, the VEF model also possesses some desirable properties of the GVF model, such as large capture range and U-shape concavity convergence. However, since *K*(*x*,*y*) is linear, the VEF model performs not very well on preserving weak edge, and is not robust to noise.

### 2.4. CONVEF: Convolutional Virtual Electric Field [31]

In a departure from the convolution in Eq (7), Wang etc. employed another convolution kernel as follows [31],

$$E_{CONVEF}=\left(-\frac{x}{d_{h}^{n+2}}⊗f,-\frac{y}{d_{h}^{n+2}}⊗f\right).$$ (9)

where $d_{h}=\sqrt{x^{2}+y^{2}+h}$. On one hand, the factor *h* plays a role analogous to scale space filtering, the larger the value of *h*, the greater the smoothing effect on the results, as a result of which the CONVEF snakes would be more robust to noise. On the other hand, the larger the value of *n*, the faster the potential decays with distance and vice versa, this property allows the CONVEF snakes to preserve weak edges and to tell apart two closely-neighboring objects with large *n*. Although large n can make the CONVEF snake preserving weak edges to some degree, the convolution kernel in Eq (9) is still linear, and there is still room for improvement.

## 3. The proposed MCONVEF model

In our proposed MCONVEF model, we set n = 1 in Eq (9) and introduce an edge stopping function *g*_*k*_(⋅) to preserve weak edges as follows,

$$E_{BCONVEF}\left(x_{0},y_{0}\right)=\sum_{\left(x,y\right)\in D}\left(\left(x_{0}-x\right)/d_{h}^{3},\left(y_{0}-y\right)/d_{h}^{3}\right)\cdot g_{k}\left(\left|\nabla f\right|\right)\cdot f\left(x,y\right)$$ (10)

where $g_{k}\left(\left|\nabla f\right|\right)=1/\left(1+{\left(\left|f\left(x,y\right)-f\left(x_{0},y_{0}\right)\right|/k\right)}^{2}\right)$. The function *g*_*k*_(|∇*f*|) has been widely employed in the anisotropic diffusion as edge stopping function to reduce the diffusion amount around the edges so that the anisotropic diffusion can preserve edges [32]. The introduction of *g*_*k*_(|∇*f*|) is expected to make the MCONVEF preserving weak edges. For points across edges, |∇*f*| is large, and then *g*_*k*_(|∇*f*|) is small, this means the points across edges will exert small impact on the weighted sum in (10), and the edges will not be smoothed out. The parameter *k* determines the contrast of the edges to be preserved. When *k* increases gradually, *g*_*k*_(|∇*f*|) increases gradually and finally approaches 1, then the proposed MCONVEF model becomes the CONVEF model with n = 1.

Since the introduction of *g*_*k*_(|∇*f*|), (10) is nonlinear and cannot be implemented using FFT directly, however, a brute-force implementation is very slow when the region D is large, therefore, we borrow the piecewise constant approximation algorithm proposed by Durant and Dorsey [33]. Observing that if *f*(*x*_0_,*y*_0_) is constant, (10) can be implemented by using the FFT, Durand and Dorsey suggested to sample *f*(*x*,*y*) into *L* segments, i.e., *f*^*j*^, j = 1,…,*L*, then, for each *f*^*j*^, the $E_{MCONVEF}^{f^{j}}$ is obtained using FFT, and the final **E**_*MCONVEF*_ is generated by linear interpolation. The implementation details are summarized in Algorithm 1. Since the calculation of **E**_*MCONVEF*_ includes *L* times FFT and a linear interpolation, the computation burden of the MCONVEF will be slightly larger than *L* times of that of the CONVEF.

**Table:** Algorithm 1. Implementation of the MCONVEF Model.

Input	image feature *f*, size of region D,segments *L*, *k* in *g*(|∇*f*|), and *h* in *d*_*h*_
Output	EMCONVEF
Initialization	j = 0; minf = min(*f*); maxf = max(*f*); deltaf = (maxf—minf)/(L-1); EMCONVEF = (0,0); $x/d_{h}^{3}\left(x,y\right)=x/{\left(\sqrt{x^{2}+y^{2}+h}\right)}^{3}$ on region D; $y/d_{h}^{3}\left(x,y\right)=y/{\left(\sqrt{x^{2}+y^{2}+h}\right)}^{3}$ on region D;
While j < L do	
*f*^*j*+1^ = minf + deltaf*j;	
*G*^*j*+1^ = *g*_*k*_(*f*−*f*^*j*+1^);	
*H*^*j*+1^ = *G*^*j*+1^×*f*;	
$E_{BCONVEF}^{f^{j+1}}=\left(-\frac{x}{d_{h}^{3}}⊗H^{j+1},-\frac{y}{d_{h}^{3}}⊗H^{j+1}\right)$/* ⊗ Convolution on region D*/	
*j = j + 1*;	
End $E_{BCONVEF}=E_{BCONVEF}+E_{BCONVEF}^{f^{j}}*InterpolationWeight(f,f^{j})$	

**Fig 1:**
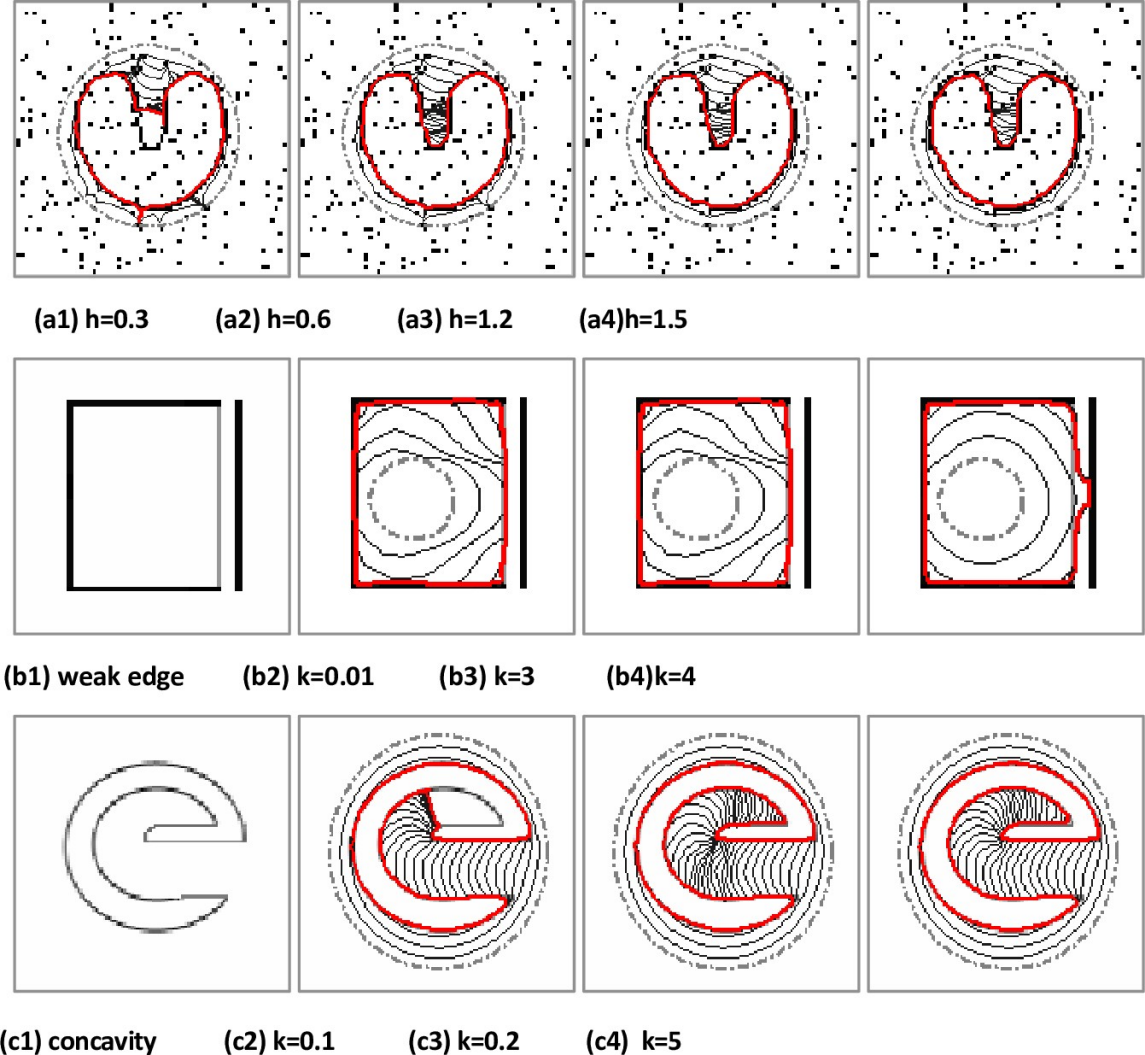
Analysis of parameter sensitivity. The gray dashed, black and red solid lines represent initialization, curve evolution and final result, respectively.

In the proposed MCONVEF model, the parameters h and k play important role in suppressing noise and preserving weak edges, respectively. Fig 1(A) shows the effect of h on the U-shape image corrupted with 10% salt and pepper noise, in this example, k = 100 so that *g*_*k*_(|∇*f*|)≈1 and the results are not influenced by the parameter k. It can be seen that the proposed MCONVEF model successfully converges to the object boundary when the parameter h varies from 0.6 to 1.5. The smaller the value of k, the weaker the edge to be preserved. Fig 1(B) shows that the k parameter ranges from 0.01 to 3, the MCONVEF model can adequately protect the weak edge near the strong one. Parameter h is zero for this example. As shown in Fig 1(C), the proposed MCONVEF model can successfully converge to the e-shape concavity with the parameter k ranging from 0.2 to 5. Since the images in Fig 1 are binary, we employed L = 2 in these experiments.

## 4. Experiments

**Fig 2:**
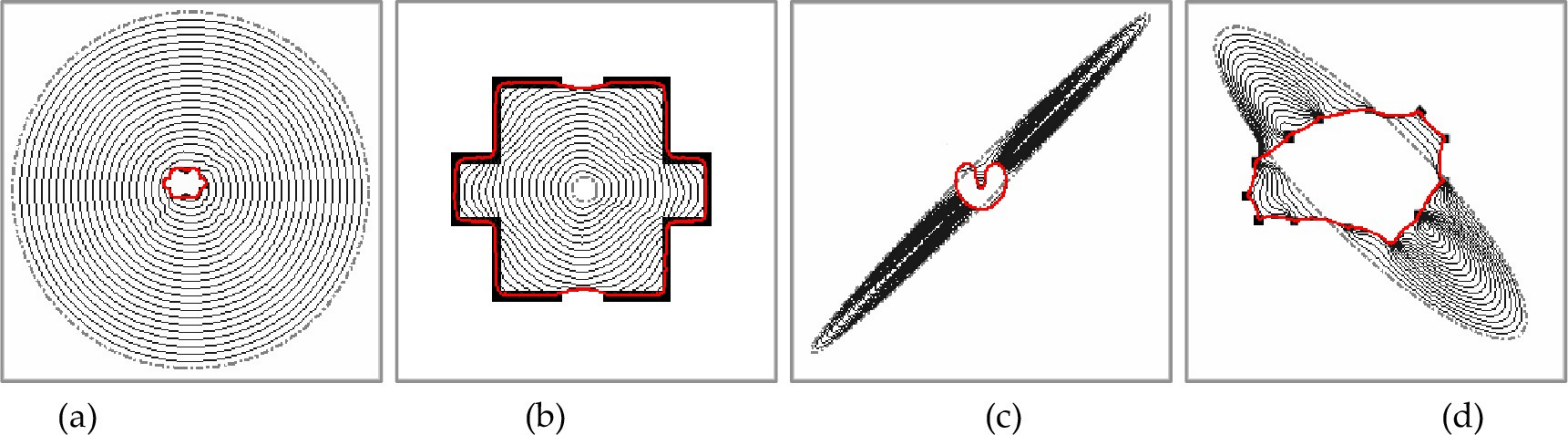
Some common properties of the MCONVEF Snakes: Large capture range, initialization insensitivity, and subject contour connection.

**Fig 3:**
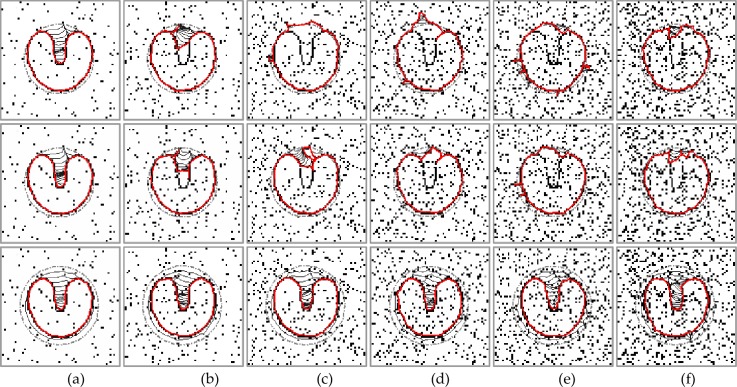
Noise resistance. U-shape images corrupted with varying salt-and-pepper noises that are (a) 0.05, (b) 0.10, (c) 0.15, (d) 0.2, (e) 0.25, (f) 0.3. First row: results of the GVF snake with μ = 0.2. Second row: results of the VEF snake. Third row: results of the MCONVEF snake with h = 1.2, k = 100, L = 2. To note when the value of the parameter k is 100, *g*_*k*_(|∇*f*|) approaches 1, and the parameter h plays a primary role in noise resistance.

**Fig 4:**
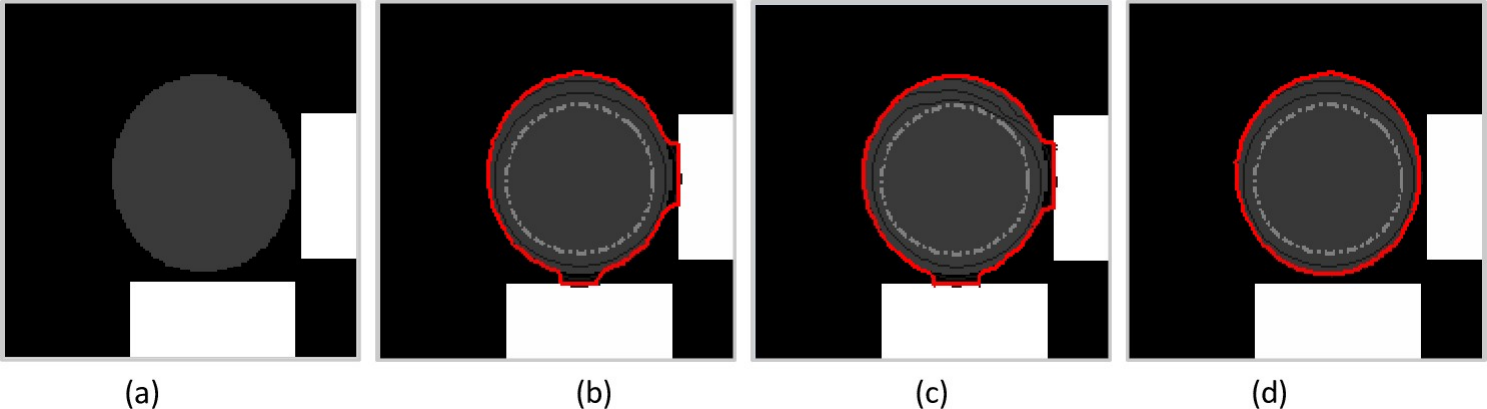
Experiment on weak edge preservation. (a) Original image. The segmentation results of (b) the GVF snake with μ = 0.05, (c) the VEF snake, and (d) the MCONVEF snake with k = 0.1, h = 0 and L = 2. The gray dash point circles are the initial contours, and the solid lines in red are the converged results.

**Fig 5:**
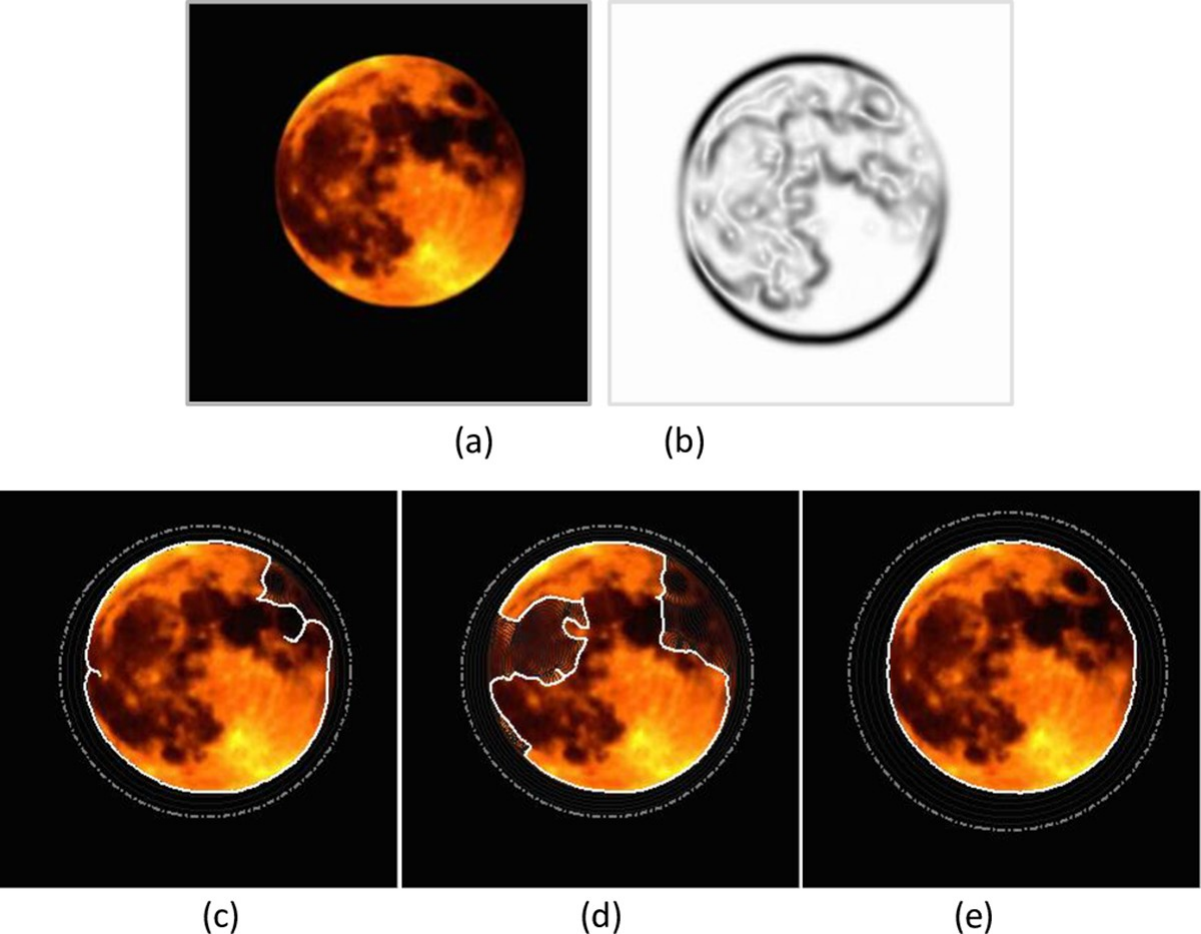
Weak edge preservation example. (a) The moon image,(b) edge map, segmentation results of (c) the GVF snake with μ = 0.05, (d) the VEF snake, and (d) the MCONVEF snake with k = 0.01, h = 0 and L = 10. The gray dash point circles are the initial contours, and the solid lines in white are the converged results.

**Fig 6:**
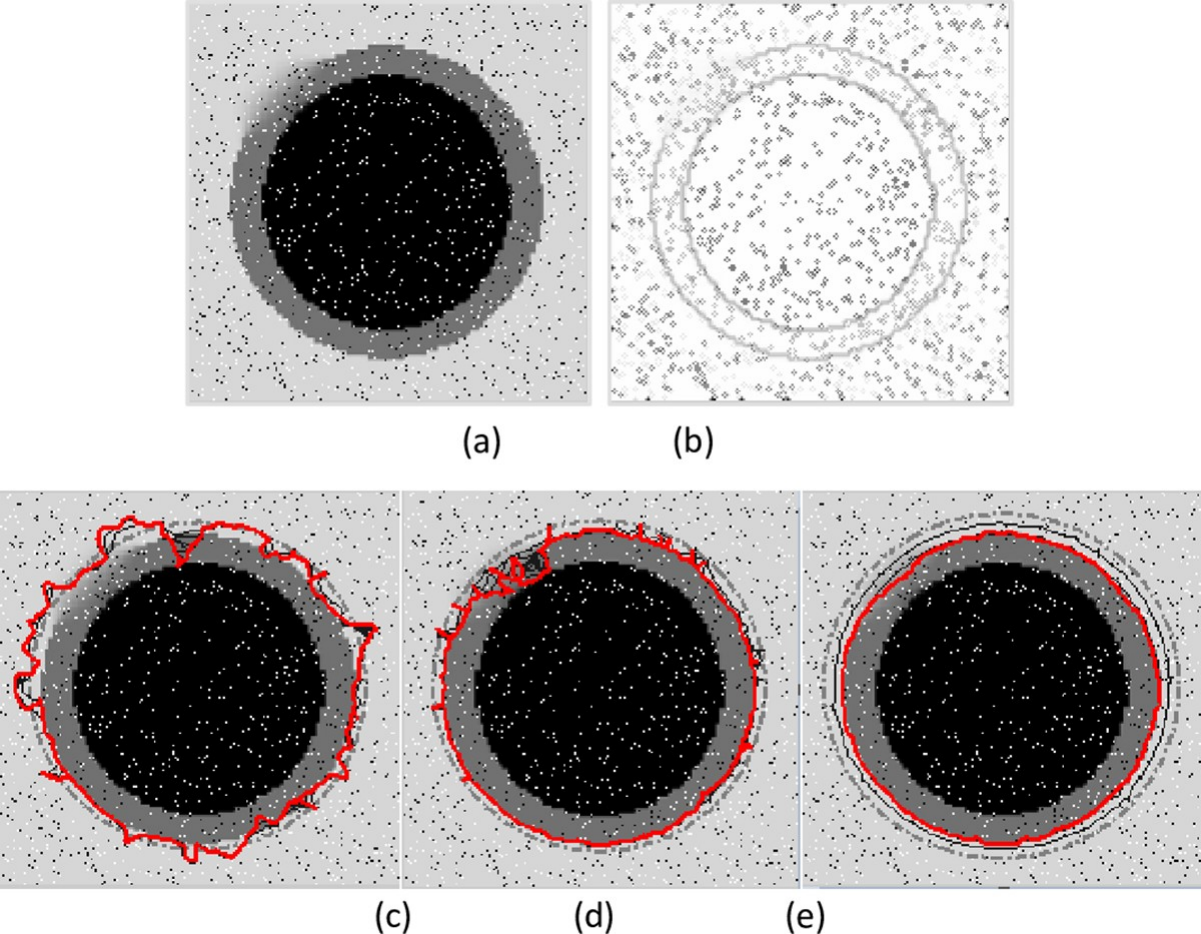
Example of weak edge preservation and noise suppression. (a) The noisy image,(b) edge map, segmentation results of (c) the GVF snake with μ = 0.1, (d) the VEF snake, and (d) the MCONVEF snake with k = 0.1, h = 1.2 and L = 2. The gray dash point circles are the initial contours, and the solid lines in red are the converged results.

**Fig 7:**
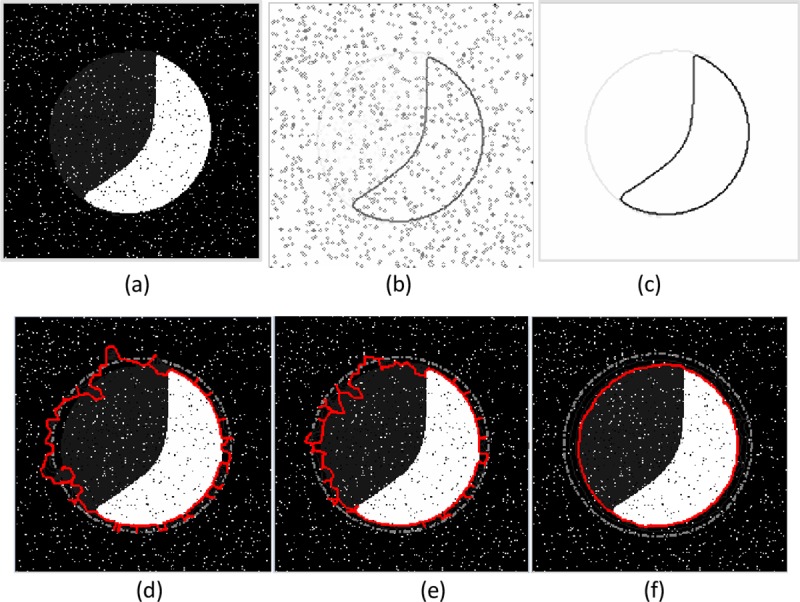
Example of weak edge preservation and noise suppression. (a) The crescent image,(b) edge map with noise, (c) edge map without noise, segmentation results of (d) the GVF snake with μ = 0.1, (e) the VEF snake, and (f) the MCONVEF snake with k = 0.1, h = 1.2 and L = 2. The gray dash point circles are the initial contours, and the solid lines in red are the converged results.

**Fig 8:**
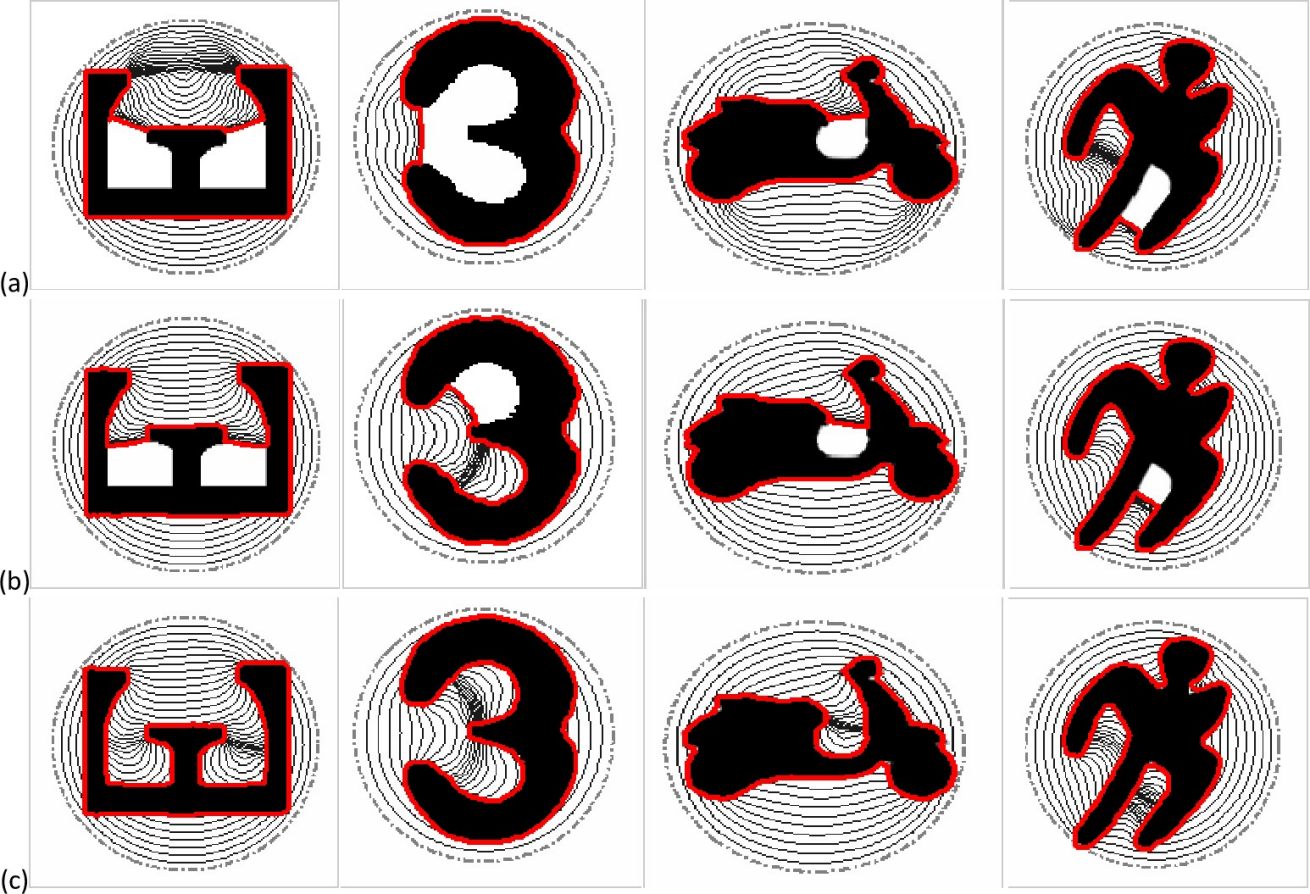
Deep concavity convergence. The segmentation results of (a) the GVF snakes with μ = 0.2, (b) the VEF snakes, and (c) the MCONVEF snakes with h = 0, k = 0.2, L = 2.

**Fig 9:**
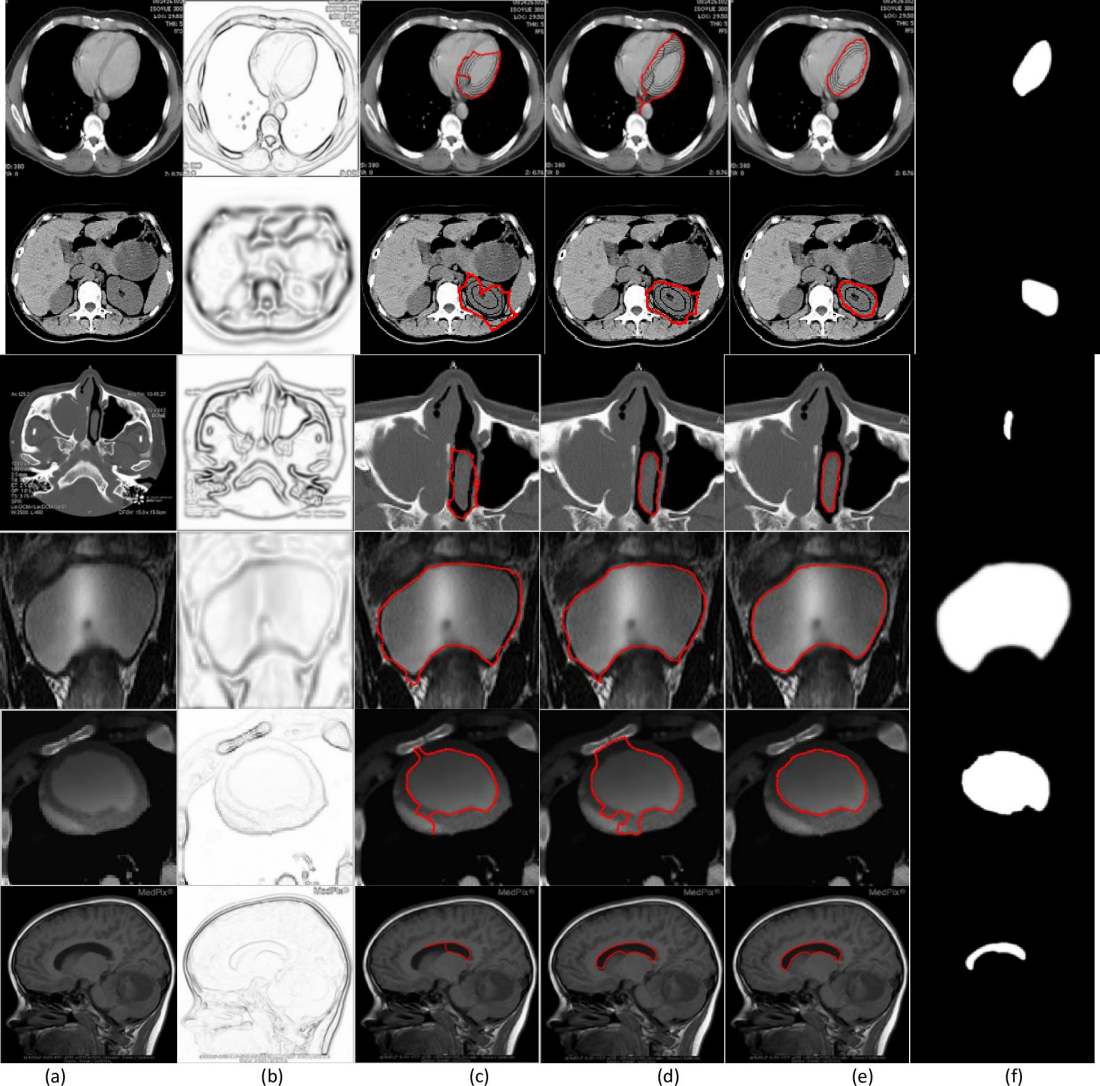
Real medical images. (a) medical images, (b) edge maps, The segmentation results of (c) the GVF snakes with μ = 0.1, (d) the VEF snakes, and (e) the MCONVEF snakes with h = 0.3, k = 0.1, L = 10, (f) ground truth.

In this section, we will demonstrate the properties of the MCONVEF snake and make comparison with the GVF and VEF snakes. The following snake parameters were used in all of our experiments, α = 0.5, β = 0.5, time step τ = 1, and the size of the convolution kernel is always set to N/2*N/2 for an image of size N*N. The image intensity is normalized to range [0,1]. Some images in experiments are coined by us, e.g., those in Figs 1,3,4,6,7 and 8, the images in Fig 2 are available at http://iacl.ece.jhu.edu/index.php/Resources#GVF_Software, the blood moon image in Fig 5 is taken by us in 2018, and the medical images in Fig 9 are provided by the General Hospital of Tianjin Medical University, and these images are obtained during routine care, the authors did not have access to any information which could be used to identify individual patients.

### 4.1. Common properties: Enlarged capture range, initial insensitivity and subject contour connection

In this experiment, a U-shape image, a room image and a subject contour image are used to verify that the proposed MCONVEF snake possesses some common and desired properties of the GVF and VEF snakes. As seen in Fig 2(A) and 2(B), the MCONVEF snakes succeed in extracting object boundaries though the initial contour is far from the target, showing that the MCONVEF snake has a large capture range. In Fig 2(C) and 2(D), the MCONVEF snakes converge successfully to target boundaries regardless of the contour initialization being across the boundary. Finally, Fig 2(D) confirms that the MCONVEF snake can connect the subject contour automatically.

### 4.2. Noise robustness

In this subsection, we evaluate the proposed MCONVEF snake model using the U-shape image corrupted with salt-and-pepper noise varying from 5% to 30%. For the proposed MCONVEF snake, the parameter *h* plays an important role in noise resistance. A more regular and smooth field can be achieved by increasing the value of h, h is set to 1.2 for this experiment. The values of parameters k and L are identical to those used in Fig 1(A). For the GVF snake, the parameter μ should be increased as the noise level increases thus μ = 0.2 was chosen. It can be seen from Fig 3 that all methods could effectively extract the target boundary when the noise level is 5%. However, when the noise variance increases, both the GVF and VEF snakes were attracted by the impulse noise. Yet, the MCONVEF snake converged to the target boundary in all cases. It should be pointed that the initial contours of the GVF and VEF snakes have been set closer to the target boundary when compared with that of the MCONVEF snake.

### 4.3. Weak edge preservation

In this subsection, we conduct several experiments to evaluate the weak edge protection performance of the MCONVEF snake model. To note, the smoothness term of the GVF model suppresses noise, but it also attenuates weak edges, one can prevent a possible weak edge leakage by reducing the value of μ. In contrast, since the MCONVEF model entails an edge stop function *g*_*k*_(|∇*f*|), one can preserve weak edge by reducing the value of k.

The gray disk in Fig 4(A) represents the target with weak edges, and the two white boxes represent the disturbing objects with strong edges. The results in Fig 4(B) and 4(C) show that the contours of the GVF and VEF snakes are attracted by the strong edges of the two white boxes. On the contrary, the MCONVEF snake can preserve the weak edge and yields a satisfactory segmentation result, see Fig 4(D). Fig 5(A) shows a moon image with a blurred silhouette on a day of total lunar eclipse. In such case, it is challenging to segment the correct target since the contrast around the boundary is too low, both the GVF and VEF snakes are attracted by edges within the moon, as shown in Fig 5(C) and Fig 5(D), respectively. For the MCONVEF snake model, it stops exactly at the target contour, see Fig 5(E).

The two examples in Figs 6~7 show the performance of the MCONVEF snake model on noise suppression and weak edge preservation simultaneously. In order to get a good balance on suppressing noise and preserving weak edges, the μ for GVF is 0.1. The image in Fig 6(A) encompasses a vague edge at the upper left, with the inner black circle yielding strong edges. The image was corrupted by salt-and-pepper noise. Fig 6(C) shows the result by the GVF snake, from which one can see the GVF snake contour leaked out from the top of black disc and even was trapped by the noise. The result of the VEF snake in Fig 6(D) is slightly better than that of the GVF snake on noise resistance, but it cannot characterize the boundary of the gray disc as well. On the contrary, the MCONVEF snake effectively smoothed out the noise and protected the weak edge simultaneously, and converged to the target successfully as shown in Fig 6(E). Another example in Fig 7(A) is a crescent image with very weak edge on the left part to compose a circle. One can discern the circle in Fig 7(C) but almost cannot discern it in the noisy edge map in Fig 7(B). Both the GVF and VEF snakes could not detect the weak edge and were attracted by noise, as illustrated in Fig 7(D) and Fig 7(E). On the contrary, the MCONVEF model performs well in converging to the target contour as shown in Fig 7(F).

### 4.4. Deep concavity convergence

In Fig 8, there are four images with various complex concavities employed to test the performance of the proposed method. The first column shows an E-shape image, and one can see that both the GVF and VEF snakes just stopped around the protuberance within the E-shape. However, the MCONVEF snake can pull the contour down to the bottom of the concavity. The second column is a 3-shape image and the GVF snake stopped at the entrance, while the VEF snake entered the low concavity but failed at the upper one. On the other hand, the MCONVEF snake succeeded in converging to the 3-shaped boundary. The results on the motorbike-shape image and the person-shape image can be seen in the third and fourth column, respectively. The overall results in these images manifest that the MCONVEF snake outperforms the other two ones in converging to complex concaves.

### 4.5. Real medical image and quantitative analysis

The segmentation of a real medical image is more challenging due to intensity inhomogeneity, noise and weak edges. In Fig 9, we employed six medical images as test, and the results are presented. From which one can see that the GVF and VEF snakes are trapped or leaked out whilst the proposed MCONVEF snake can get satisfactory results. In contrast to the previous experiments, we present the ground truth at the last column, and we adopt Precision, Recall, and F1 measures [2] as three objective metrics in our experiment, more details on these three indices can be referred to ref.[2]. The quantitative results are reported in Table 1, it can be seen from Table 1 that the MCONVEF snake yields the most excellent Precision and F1-measure, which means the MCONVEF snake yields the most excellent segmentation results.

**Table 1:** The comparison of quantitative indices on medical images.

Image	GVF			VEF			MCONVEF		
	Precision	Recall	F1 measure	Precision	Recall	F1 measure	Precision	Recall	F1 measure
mediastinum	0.7733	0.8482	0.8090	0.7533	1	0.8593	0.9477	0.9949	0.9707
pancreatic	0.5527	0.9364	0.6951	0.6703	0.9679	0.7918	0.9676	0.9500	0.9587
nasal bone	0.5837	0.9210	0.7145	0.8404	0.9396	0.8872	0.9581	0.9160	0.9369
knee joint	0.8621	0.9945	0.9235	0.9367	0.9840	0.9597	0.9837	0.9822	0.9830
cardiac	0.9047	0.9934	0.9469	0.7849	0.9952	0.8776	0.9378	0.9930	0.9646
brain	0.9851	0.4737	0.6398	0.9472	0.9419	0.9445	0.9466	0.9737	0.9600
Average	0.7855	0.8794	0.8050	0.7772	0.9737	0.8559	0.9454	0.9701	0.9571

### 4.6. Computational cost

In order to make comparison of the runtime of the proposed MCONVEF model and that of the GVF and VEF models, we coined several line-drawing images with the size varying from 64*64 to 512*512. Since the computational cost of both the MCONVEF and VEF model depends on the size of the convolution kernel, the convolution kernels are of size N/2*N/2, and for the GVF model, the iteration number is N for an image of size N*N. Table 2 presents the corresponding runtime in seconds, it can be seen that the computation cost of the MCONVEF model is much less than that of the GVF model, and slightly higher than L times of that of the VEF model due to the interpolation.

**Table 2:** Runtime comparison of the MCONVEF, GVF and VEF models in second.

	N = 64	N = 128	N = 256	N = 512
GVF	0.23	2.22	27.60	424.04
VEF	0.01	0.07	0.52	1.86
MCONVEF(L = 2)	0.02	0.14	1.08	3.83
MCONVEF(L = 10)	0.12	0.71	5.20	18.86

## 5. Conclusions

In this paper, we proposed a novel external force for active contours, namely, the MCONVEF model. The proposed MCONVEF model introduced the scale-space parameter h and the edge stopping function *g*_*k*_(|∇*f*|) and employed a piecewise linear approximation to achieve fast calculation. Experimental results on both synthetic and real images have shown that the MCONVEF snake model holds the desirable properties of the GVF and VEF snakes such as the large capture range, initialization insensitivity, and subject contour convergence. Additionally, the proposed model presents better performance in quantitative metrics in terms of noise robustness, weak edge protection and deep concavity convergence. In summary, the MCONVEF model can be considered as a superior alternative to the GVF and VEF models.
